# Advances in understanding cold tolerance in grapevine

**DOI:** 10.1093/plphys/kiad092

**Published:** 2023-02-15

**Authors:** Chong Ren, Peige Fan, Shaohua Li, Zhenchang Liang

**Affiliations:** Beijing Key Laboratory of Grape Sciences and Enology, Key Laboratory of Plant Resource, Institute of Botany, Chinese Academy of Sciences, Beijing 100093, PR China; China National Botanical Garden, Beijing 100093, PR China; Beijing Key Laboratory of Grape Sciences and Enology, Key Laboratory of Plant Resource, Institute of Botany, Chinese Academy of Sciences, Beijing 100093, PR China; China National Botanical Garden, Beijing 100093, PR China; Beijing Key Laboratory of Grape Sciences and Enology, Key Laboratory of Plant Resource, Institute of Botany, Chinese Academy of Sciences, Beijing 100093, PR China; China National Botanical Garden, Beijing 100093, PR China; Beijing Key Laboratory of Grape Sciences and Enology, Key Laboratory of Plant Resource, Institute of Botany, Chinese Academy of Sciences, Beijing 100093, PR China; China National Botanical Garden, Beijing 100093, PR China

## Abstract

Grapevine (*Vitis* ssp.) is a deciduous perennial fruit crop, and the canes and buds of grapevine should withstand low temperatures (LTs) annually during winter. However, the widely cultivated *Vitis vinifera* is cold-sensitive and cannot survive the severe winter in regions with extremely LTs, such as viticulture regions in northern China. By contrast, a few wild *Vitis* species like *V. amurensis* and *V. riparia* exhibit excellent freezing tolerance. However, the mechanisms underlying grapevine cold tolerance remain largely unknown. In recent years, much progress has been made in elucidating the mechanisms, owing to the advances in sequencing and molecular biotechnology. Assembly of grapevine genomes together with resequencing and transcriptome data enable researchers to conduct genomic and transcriptomic analyses in various grapevine genotypes and populations to explore genetic variations involved in cold tolerance. In addition, a number of pivotal genes have been identified and functionally characterized. In this review, we summarize recent major advances in physiological and molecular analyses of cold tolerance in grapevine and put forward questions in this field. We also discuss the strategies for improving the tolerance of grapevine to cold stress. Understanding grapevine cold tolerance will facilitate the development of grapevines for adaption to global climate change.

## Teaser

A comprehensive review summarizes recent advances in knowledge of cold tolerance in grapevine.

## Introduction

Cold stress, including chilling (0 °C to 15 °C) and freezing (<0 °C) stresses, has an adverse effect on plant growth, development, productivity, and geographical distribution (Thomashow 1999; Ding et al. 2019). Chilling stress affects the composition of membrane lipids, decreases the activities of intracellular enzymes, attenuates the stability of protein complexes, and impairs photosynthesis (Siddiqui and Cavicchioli 2006; Ruelland et al. 2009). Freezing stress usually results in formation of ice crystals in the apoplast, which induces cell dehydration due to the efflux of water (Ritonga and Chen 2020). When plant cells are filled with ice crystals, cell membranes are destroyed, resulting in cell death. Temperate plants have evolved with the ability to withstand freezing stress through a process called cold acclimation (CA) after exposure to low nonfreezing temperatures for a few days (Thomashow 1999). CA induces an array of physiological and biochemical changes involving transcriptional regulation of *COLD REGULATED* (*COR*) genes. Up- or down-regulation of *COR* genes affects the abundance of phytohormones, metabolites, and specific proteins (Maruyama et al. 2012; Nakaminami et al. 2014).

**Advances Box**

- Cold stress induces the accumulation of phytohormones (e.g. ABA and ethylene) and metabolites (e.g. proline and soluble sugars), which in turn modulate the tolerance of grapevine to cold stress.
- The CBF-dependent signaling pathway in grapevine is rapidly triggered by cold and functions as a principal regulatory network in cold response.
- Grapevine ncRNAs, particularly lncRNAs and miRNAs, function as important regulators in cold-responsive regulatory networks by targeting cold-related genes.
- Assembly of the grapevine genome together with resequencing has provided a genomic basis for mining cold-tolerant genes.

**Figure 1.**
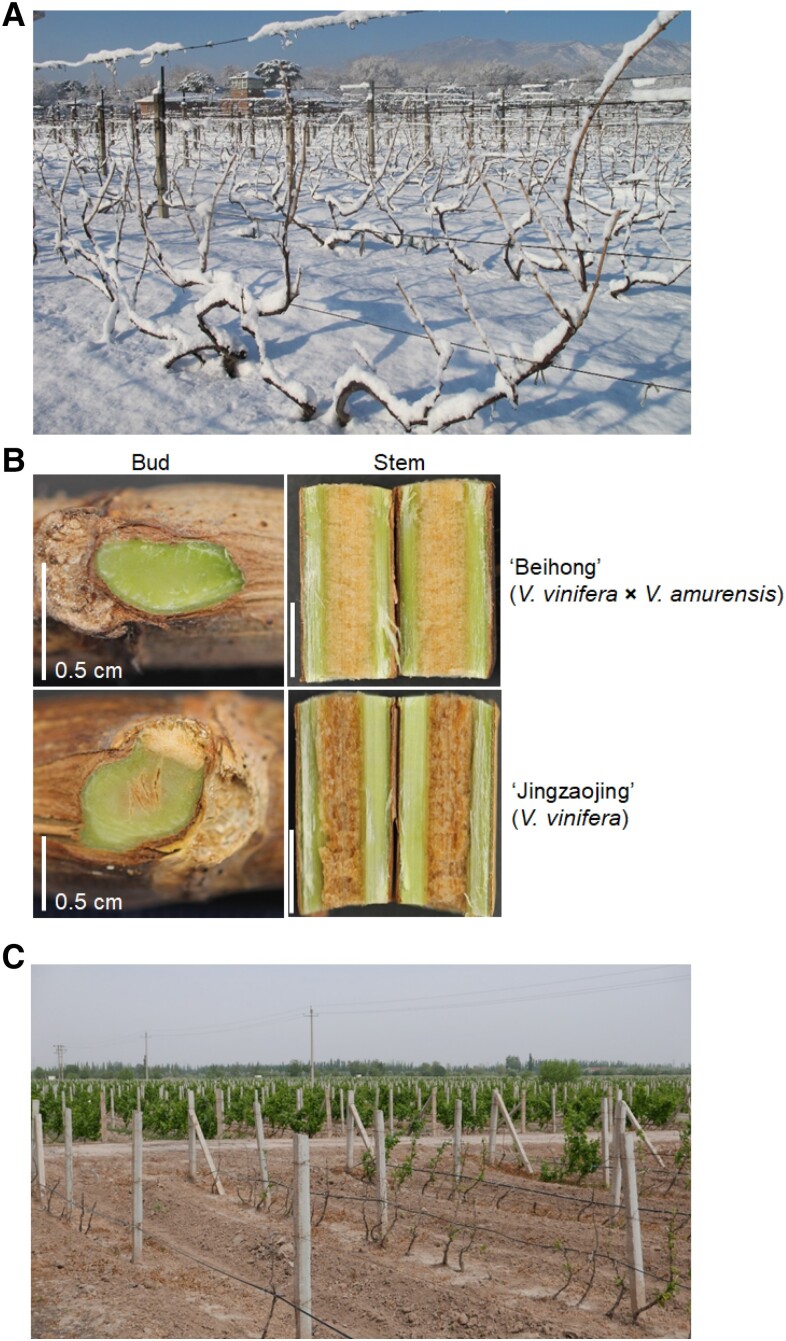
The influence of cold stress on grapevine. **A)** The grapevines cultivated in Beijing, e.g. generally encounter freezing stress during winter. **B)** Phenotypes of grapevine buds and canes after exposure to freezing temperatures (<−5 °C). Damages were observed in buds and stems of cold-sensitive *V. vinifera* cv. Jingzaojing, while cold-tolerant grapevine “Beihong”, the hybrid of *V. vinifera* and *V. amurensis*, was detected with no obvious damages. **C)** Phenotypes of overwintering grapevines in spring in Ningxia, China. For *V. vinifera*, the vines should be buried in soil to survive the severe winter in northern China. However, the bud germination and growth of buried “Cabernet Sauvignon” (*V. vinifera*, at image lower part) are obviously affected by low winter temperatures when compared with “Beihong” (*V. vinifera × V. amurensis*, at image upper part) without burying treatment.

Grapevine (*Vitis* spp.) is a perennial fruit crop whose vines and buds should survive cold stress in winter. However, the cultivated *Vitis vinifera* cannot survive the severe winter in northern China, and the vines require burial in soil during the winter (Fig. 1). Unlike *V. vinifera* cultivars, some *Vitis* species from wild grape germplasm show excellent tolerance to low temperatures (LTs). The cold-hardy species *V. amurensis*, which originated from eastern Asia, tolerates temperatures as low as −40 °C (Fennell 2004). Many *V. amurensis* accessions such as “Zuoshan-1” and “Heilongjiang” have been utilized as breeding materials in China. *Vitis riparia*, the native American grapevine species, has been used extensively in rootstock and scion breeding for its freezing tolerance (Luby and Fennell 2006). However, the underlying mechanisms with respect to cold tolerance in grapevine are still largely unknown. In recent years, increasing studies have uncovered possible mechanisms involved in cold tolerance at the physiological and molecular levels in grapevine. In this review, we summarize recent advances in cold response in grapevine, aiming to provide a comprehensive overview of our current understanding of cold signaling in this species.

## Cold sensing

To adapt to LTs, plants need to perceive cold stimulus and convey cold signaling. The decreased plasma membrane (PM) fluidity is thought to be an important cold-sensing mechanism. Pharmacological studies showed that *COR* expression could be induced by membrane rigidification independent of temperatures (Orvar et al. 2000; Sangwan et al. 2001). Many PM-localized proteins, such as calcium channels, G-protein associated receptors, and receptor-like kinases (RLKs), have also been identified as cold sensors. Cold stress could induce rapid Ca^2+^ influx into the cytosol through calcium channels (Wilkins et al. 2016; Mori et al. 2018). The PM- and endoplasmic reticulum-localized G-protein regulator CHILLING TOLERANCE DIVERGENCE1 has recently been identified as a cold sensor in rice (*Oryza sativa*) to mediate cold-induced influx of Ca^2+^ by interacting with RICE G-PROTEIN α SUBUNIT1 (Ma et al. 2015).

**Figure 2.**
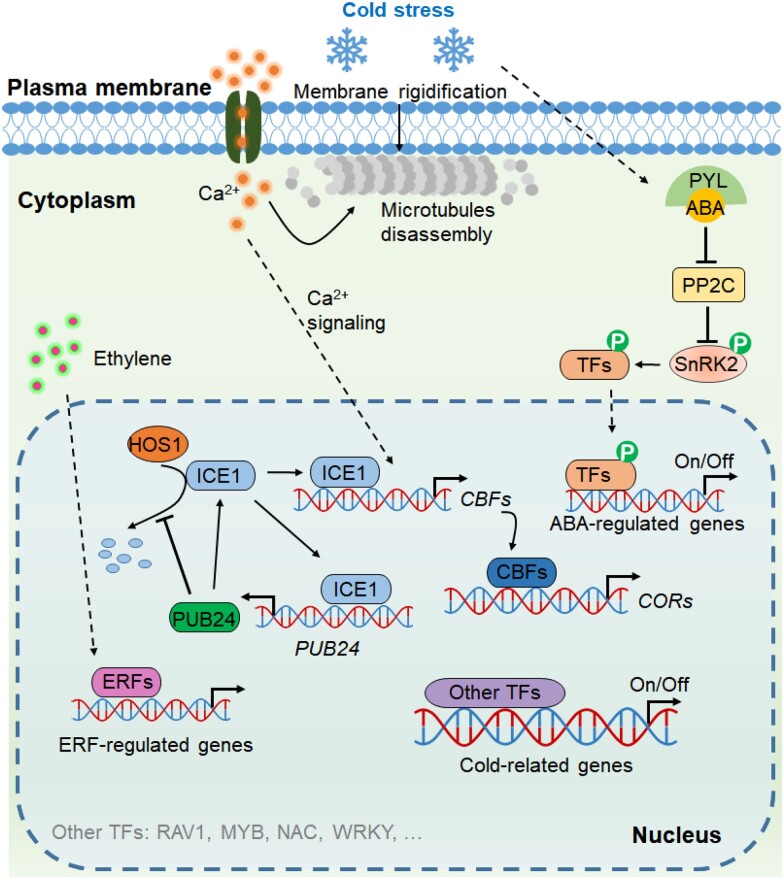
Cold perception and transcriptional regulation of cold-related genes in grapevine. Cold stress induces membrane rigidification and Ca^2+^ influx, which are necessary for microtubule disassembly. The disassembly of microtubules acts as a cold-sensing event that amplifies cold signaling. Upon cold stress, ICE1 activates *CBFs* expression, which promote the expression of downstream *COR* genes. Notably, the influx of Ca^2+^ is necessary for cold activation of *CBF4*. Furthermore, ICE1 could also promote the expression of *PUB24* (*plant U-box protein 24*), and PUB24 competes with HOS1 (HIGH EXPRESSION OF OSMOTICALLY RESPONSIVE GENE 1, E3 ubiquitin ligase) for binding ICE1, thereby promoting the accumulation of ICE1. Moreover, cold-induced ABA bound by its receptor PYL (PYRABACTIN RESISTANCE-LIKE) could release SnRK2 from the PP2C (PROTEIN PHOSPHATASE 2C)–SnRK2 complex, and the released SnRK2 phosphorylates-specific TFs, which regulate the expression of ABA-regulated genes. Similarly, cold-induced ethylene may regulate downstream genes by affecting the activities of ERFs through ethylene signaling pathway. In addition, other TFs such as MYB, NAC, and WRKY also contribute to cold tolerance in grapevine by regulating their own target genes.

Plant cytoskeleton, including microtubules and actin filaments, is involved in perception of external stress and signal transduction as well (Nick 2013; Lian et al. 2021). The relative rigidity of microtubules influences membrane fluidity, thereby affecting cytosolic Ca^2+^ concentration (Wang and Nick 2017; Wang et al. 2021a). In grape cells, by using green fluorescent protein fusions of Arabidopsis (*Arabidopsis thaliana*) tubulin as the reporter, microtubules are found to disappear within 30 min after exposure to cold stress; the Ca^2+^ influx, membrane rigidification, and NADPH oxidase activity are necessary for cold-induced microtubule disassembly, which may act as a sensory event to amplify cold signal (Wang and Nick 2017) (Fig. 2). Furthermore, microtubule stability could modulate cold-signaling sensitivity in grape cells (Wang et al. 2019a). Microtubules together with membrane fluidity are regarded as upstream factors of cold sensing and signaling, and the role of microtubules in cold signaling has been comprehensively reviewed recently (Wang et al. 2020d; Kumar et al. 2022).

## Physiological changes in response to cold stress in grapevine

**Figure 3.**
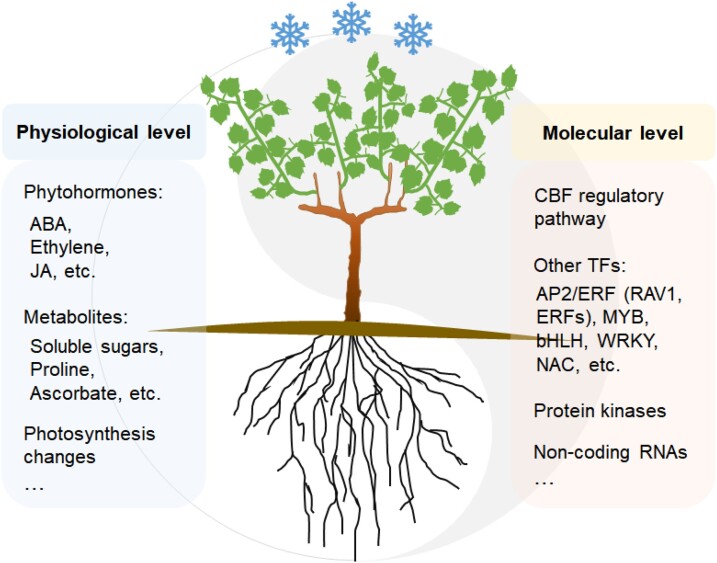
Overview of physiological and molecular changes in response to cold stress in grapevine. After exposure to cold stress, grapevine plants accumulate phytohormones (e.g. ABA, ethylene, JA, etc.) and metabolites (e.g. soluble sugars, proline, ascorbate, etc.), which can trigger hormone-related regulatory networks or function as osmolytes and cryoprotectants by inhibiting freezing-induced dehydration and scavenging reactive oxygen species. Photosynthesis is also changed to adapt to cold stress. At the molecular level, both CBF-dependent and independent (other TFs) pathways are activated to regulate the cold tolerance of grapevine. Protein kinases and noncoding RNAs play crucial roles in cold response as well. The changes happened in grapevine at the physiological and molecular levels are tightly associated. Transcriptional regulation of cold-related genes results in physiological changes, and dynamic changes of metabolites in turn affect genes expression. In sum, grapevine plants orchestrate physiological and molecular changes to regulate its plasticity to cope with external cold stress.

After exposure to cold stress, the cold signal is perceived and transduced into cells, triggering a series of transcriptional, translational, and metabolic changes. The altered levels of phytohormones and metabolites (Fig. 3) confer increased cold tolerance on plants.

### Phytohormones

Abscisic acid (ABA) is a stress-response phytohormone known for its crucial roles in response to abiotic stresses, such as drought, high salinity, and cold stress (Umezawa et al. 2020). For grapevine buds, cold hardiness is closely associated with bud dormancy, which is affected by temperature fluctuations throughout the dormant season (Ferguson et al. 2011; Kovaleski et al. 2018). The cold hardiness of grapevine buds has been evaluated using a dynamic predictive model (Ferguson et al. 2011). Interestingly, the ABA level in grapevine buds gradually increases in autumn, and the accumulated ABA regulates bud dormancy and protects buds from LTs in winter (Zheng et al. 2015; Rubio et al. 2019a). Foliar application of ABA advances grapevine bud dormancy and increases the bud freezing tolerance (Zhang and Dami 2012; Dami et al. 2015; Li and Dami 2016; Wang et al. 202°c). Exogenous ABA treatment decreases grapevine bud water content and induces accumulation of stachyose, myo-inositol, sucrose, raffinose, and galactinol; the expression of *raffinose synthase 1* (*RafS1*), *galactinol synthase 1* (*GolS1*), and *GolS2* genes is accordingly upregulated (Zhang and Dami 2012; Wang et al. 202°c). The activities of antioxidant enzymes and the scavenging of reactive oxygen species are enhanced as well (Wang et al. 202°c). Notably, the effect of exogenous ABA treatment is influenced by vine phenological stages, with veraison and postveraison being the best stages for ABA treatment (Dami et al. 2015; Li and Dami 2016). Importantly, ABA-induced transcriptomic changes have been found to be modulated by several negative feedback systems in grapevine, which may help to reduce long-term negative effects of exogenous ABA on grapevine growth (Wang et al. 2022). In the past decades, the members of the ABA-signaling pathway have been widely identified and studied in many plants. In grapevine, there are 9 *PYRABACTIN RESISTANCE-LIKE* (*VvPYL*), 85 *PROTEIN PHOSPHATASE 2C* (*VvPP2C*), 7 *SNF1-RELATED PROTEIN KINASE 2* (*VvSnRK2*), and 8 *ABA-RESPONSIVE ELEMENT BINDING FACTOR* (*VvABF*) genes, among which *VvPP2C59*, *VvPP2C60*, *VvPP2C66*, and *VvABF8* were found to be involved in cold response (Zhang et al. 2021). Recently, *V. amurensis PYL1* (*VaPYL1*), *VaPYL4*, *VaPYL5*, and *VaPYL13* were found to be induced by cold stress (Ren et al. 2022), and overexpression of *VaPYL4* in Arabidopsis and *VaPYL9* in tomato (*Solanum lycopersicum*) improved plant resistance to cold stress (Ren et al. 2022; Nai et al. 2022).

Ethylene is a gaseous phytohormone that participates in plant development and responses to biotic and abiotic stresses (Wang et al. 2013a; Ju and Chang 2015). However, the effect of ethylene on cold tolerance varies in different plant species. Increased ethylene levels improved cold resistance in tomato, tobacco (*Nicotiana tabacum*), mandarin (*Citrus clementina* × *Citrus reticulata*), and apple (*Malus domestica*) (Lafuente et al. 2004; Wang et al. 2021b; Zhang and Huang 2010), whereas ethylene level in *Medicago truncatula* is negatively associated with cold tolerance (Zhao et al. 2014). Chilling stress induces rapid release of ethylene in grapevine, and the application of ethylene precursor 1-aminocyclopropane-1-carboxylic acid enhances grapevine tolerance to cold stress (Sun et al. 2016). Ectopic overexpression of *V. amurensis ethylene responsive factor 057* (*VaERF057*) and *VaERF092* in Arabidopsis enhanced its freezing tolerance (Sun et al. 2016, 2019). Intriguingly, ethylene functions in dormancy release of grapevine buds (Shi et al. 2018b) indicating that ethylene and ABA act antagonistically in the regulation of bud dormancy. The crosstalk of ethylene with ABA in cold tolerance in grapevine requires further investigation.

Salicylic acid (SA) and jasmonic acid (JA) are generally associated with plant defense. Emerging evidence shows that SA is induced by LTs in Arabidopsis, wheat (*Triticum aestivum*), and grape berries (Scott et al. 2004; Wan et al. 2009; Kosová et al. 2012). Exogenous application of SA increases the expression of grape *C-REPEAT BINDING FACTOR 1* (*CBF1*), *CBF2*, *CBF3*, and *CBF4* under cold-stress conditions; the antioxidant enzyme activities and contents of soluble sugars and proline are also increased (Aazami and Mahna 2017; Li and Wang 2021). JA positively regulates plant cold tolerance by modulating the interactions between JASMONATE ZIM-DOMAIN PROTEIN 1 (JAZ1)/JAZ4 and INDUCER OF *CBF* EXPRESSION 1 (ICE1)/ICE2, thereby promoting the expression of *CBFs* in Arabidopsis (Hu et al. 2013). In grapevine, JA has been widely acknowledged as a regulator of fruit ripening, fruit-pedicel abscission, and plant defense against pathogens (Jia et al. 2016; Coelho et al. 2019; Fidelibus et al. 2022). Study of grapevine methyl esterase (MES) family revealed that *VvMES5*, which encodes a methyl esterase catalyzing the demethylation of methyl jasmonate (MeJA), was substantially induced by cold and UV-B (Zhao et al. 2016). Furthermore, the JA-signaling pathway gene *JAZ1* was upregulated after CA in grapevine (Wu et al. 2014). More recently, the *V. amurensis* phytochrome A signal transduction 1 (VaPAT1) coupled with VaIDD3 (indeterminate-domain 3) has been demonstrated to regulate JA biosynthesis by activating the jasmonate biosynthesis gene *LOX3* (*lipoxygenase 3*) in response to cold stress; treatment of grape calli with exogenous MeJA could improve its cold tolerance (Wang et al. 2021e). Moreover, JA may act as upstream signal of cold stress by triggering the disassembly of microtubules (Wang and Nick 2017).

Though previous studies mostly focus on specific phytohormones, it is worth noting that hormonal crosstalk during cold signaling is commonly observed and crucial in regulating the balance between plant growth and cold response (Waadt et al. 2022).

### Metabolites

To understand the metabolic changes during CA, Chai et al. (2019) conducted comparative metabolic analysis to detect the metabolites in both *V. amurensis* and *V. vinifera* leaves after chilling treatment. Carbohydrates, amino acids, and organic acids are commonly induced by cold stress, whereas some metabolites are differentially accumulated in different species. For instance, proline is remarkably accumulated in *V. amurensis*, while myo-inositol is substantially increased in *V. vinifera*. Moreover, galactinol, ascorbate, and putrescine are also preferentially accumulate in *V. amurensis* (Chai et al. 2019). The roles of proline, ascorbate, and putrescine in plant cold tolerance have been reported in previous studies (Li et al. 2021b; Ghosh et al. 2022; Song et al. 2022b). Galactinol is necessary for biosynthesis of raffinose family of oligosaccharides (RFOs), which act as osmolytes to protect cells from cold-induced dehydration or damage (Sengupta et al. 2015). In addition to the RFOs, soluble sugars such as fructose and sucrose are also increased in grapevine leaves upon cold stress; consistently, the genes involved in sucrose biosynthesis are highly expressed (Londo et al. 2018; Liang et al. 2022). Notably, the metabolites detected in grapevine leaves, such as sucrose and galactinol, also accumulate in grapevine buds (Table 1). In grapevine inflorescences, the content of γ-aminobutyrate, alanine, and lysine is obviously increased upon chilling stress. However, the impact of chilling stress on starch and trehalose accumulation in inflorescences depends on cultivars. For example, starch level was increased in cold-treated flowers in “Pinot noir”, while in “Gewurztraminer” the starch content was not affected (Sawicki et al. 2015).

**Table 1.** Accumulated metabolites in grapevine organs upon cold stress or ABA treatment

Organ	Treatment	Accumulated metabolites	Grapevine	Reference
Bud	Chilling and freezing	Sucrose, fructose, glucose, and raffinose (transiently increased)	“Merlot” × ”Kozma 20–3” (hybrid)	De Rosa et al. (2022)
	ABA	Sucrose, galactinol, stachyose, raffinose, and myo-inositol	“Cabernet franc” (*V. vinifera*)	Wang et al. (2020b)
Leaf	Chilling	Sucrose, glucose, fructose, maltose, raffinose, melibiose, and γ-aminobutyric acid	“Muscat hamburg” (*V. vinifera*)	Chai et al. (2019)
	Chilling	Sucrose, glucose, fructose, raffinose, galactinol, proline, putrescine, and ascorbate	*V. amurensis*	
Flower	Chilling	Glucose and fructose	“Pinot noir” (*V. vinifera*)	Sawicki et al. (2012)
	Freezing	Sucrose, fructose, and starch		
	Chilling	Sucrose, glucose, fructose, γ-aminobutyrate, alanine, and lysine	“Gewurztraminer” (*V. vinifera*)	Sawicki et al. (2015)
		Starch, γ-aminobutyrate, alanine, and lysine	“Pinot noir” (*V. vinifera*)	

### Photosynthesis

Photosynthesis is impaired by LTs through limiting stomatal aperture, thylakoid electron transport, and activities of Rubisco and key enzymes in starch and sucrose biosynthesis (Allen and Ort 2001). Cold-induced grapevine growth retardation is partially due to the inhibition of grapevine leaf photosynthesis (Hendrickson et al. 2004; Bertamini et al. 2005, 2006). Cold stress dramatically decreased the minimal fluorescence (*F*_0_), maximal fluorescence (*F*_m_), and the maximum photochemical quantum yield of photosystem II (*F*_v_/*F*_m_) (Hendrickson et al. 2004; Sawicki et al. 2012; Aazami et al. 2021). Grapevines with higher cold tolerance usually exhibit less decrease in *F*_m_ and *F*_v_/*F*_m_ (Su et al. 2015; Aazami et al. 2021). Intriguingly, the *F*_v_/*F*_m_ shows a significant linear correlation with electrolyte leakage, which is generally employed to evaluate cold tolerance in plants, in grapevines with different genotypes (Su et al. 2015). Hence, a *F*_v_/*F*_m_-based model has been developed to evaluate the tolerance of grapevines to drought-cold stress (Su et al. 2015). Study of grapevine responses to drought stress reveals two ways, namely stomatal and nonstomatal processes, that affect photosynthesis (Maroco et al. 2002). Likewise, LTs could influence grapevine photosynthesis via both stomatal and nonstomatal mechanisms, and the mechanisms are differentially induced by cold according to the stress intensity (Hendrickson et al. 2004; Sawicki et al. 2012).

## Cold-responsive regulatory networks in grapevine

Cold-induced physiological changes are generally controlled by transcriptional regulation of *COR* genes and translational modifications of specific proteins. Transcription factors (TFs) and protein kinases play pivotal roles in these processes.

### The core CBF-dependent regulatory pathway

CBFs, also known as DEHYDRATION-RESPONSIVE ELEMENT (DRE)-BINDING PROTEIN1 (DREB1), are identified as key TFs in cold response in plants (Thomashow 1999). *CBF* genes are rapidly induced by cold, and CBFs then regulate expression of *COR* genes by binding to the C-repeat (CRT)/DRE cis-element in their promoters (Thomashow 1999; Shi et al. 2018a). The members and functions of *CBFs* have been characterized in many plant species, including grapevine. Four *CBFs* were initially identified in *V. vinifera* and *V. riparia*; among which *CBF4* is strongly induced by cold, while *CBF1*, *CBF2*, and *CBF3* preferentially respond to drought stress (Xiao et al. 2006, 2008). Later, seven *CBF* genes were cloned from *V. vinifera* and *V. riparia* (Carlow et al. 2017). However, further analysis of *V. vinifera* genome revealed that *VvCBF1* and *VvCBF2* correspond to the same gene, and there are six members currently recorded in grapevine (Wisniewski et al. 2014; Vázquez-Hernandez et al. 2017; Rubio et al. 2019b). The expression of *VvCBF2*, *VvCBF3*, *VvCBF4*, and *VvCBF6* is induced by cold and ABA (Rubio et al. 2019b). Intriguingly, Ca^2+^ influx is necessary and sufficient for cold-activated *CBF4* expression in grapevine (Wang et al. 2019a) (Fig. 2). Overexpression of *CBFs* from *V. vinifera* or *V. riparia* could improve freezing tolerance in plants (Takuhara et al. 2010; Siddiqua and Nassuth 2011; Tillett et al. 2012). The CBF regulon detected in *VvCBF4*-overexpressing grapevine plant is similar to that observed in Arabidopsis and poplar (*Populus*), indicating that the CBF-regulatory pathway is relatively conserved in plants (Tillett et al. 2012).

ICE1 is a chief regulator of *CBF* genes (Chinnusamy et al. 2003; Kim et al. 2015). In Arabidopsis, ICE1 and ICE2 function redundantly in regulating *CBF1* expression (Fursova et al. 2009; Kim et al. 2015). Grape contains four *ICE* genes, which produce at least seven different ICE proteins through alternative polyadenylation (Rahman et al. 2014). *VaICE1* is strongly induced in grapevine roots, leaves, stems, and petioles by cold stress. Overexpression of *VaICE1* in tobacco improved its cold tolerance (Dong et al. 2013). Likewise, overexpressing *VaICE1* and *VaICE2* upregulated the expression of *AtCBF1*, *AtCOR15A*, and *AtCOR47*, thereby increasing the freezing tolerance of transgenic Arabidopsis (Xu et al. 2014a). Interestingly, overexpressing *VrCBF1* and *VrCBF4* in Arabidopsis can positively regulate the expression of *AtICE1* (Siddiqua and Nassuth 2011), suggesting a difference in gene regulation across different species. A recent study reported that *Vitis pseudoreticulata* ICE1 could promote the expression of *VpPUB24* (*plant U-box protein 24*) at LTs, and VpPUB24 in turn interacts with VpICE1 to promote its accumulation (Yao et al. 2017). More recently, Kidokoro et al. (2020) found that ICE1 had no effect on *CBFs* induction, and the repression of *CBFs* in the Arabidopsis *ice1* mutant was caused by DNA methylation-mediated gene silencing rather than the mutation of ICE1. The result is apparently inconsistent with previous findings (Ding et al. 2015; Kim et al. 2015; Miura et al. 2011), challenging the role of ICE1 in regulation of *CBF* genes.

In addition to ICE1, calmodulin-binding transcription activator (CAMTA) proteins are also important regulators of *CBFs* expression (Doherty et al. 2009; Kidokoro et al. 2017). A total of 10 *CAMTA* genes have been identified with tissue-specific expression patterns in grapevine, but their functions in cold response remain unknown (Shangguan et al. 2014). Accumulating evidence shows that circadian clock components REVEILLE4 (RVE4) and RVE8 could bind to the evening elements in the promoters of *CBF* genes and activate their expression (Dong et al. 2011; Kidokoro et al. 2021). However, circadian regulation of cold response in grapevine has not been reported yet. Recent studies provide evidence showing that photoreceptor phyB, pytochrome-interacting factors (PIFs) and CBFs form regulatory networks to integrate light and cold signaling in Arabidopsis (Jiang et al. 2020; Dong et al. 2020). However, the function of PIF4 in Arabidopsis and tomato is different, given that AtPIF4 represses *CBFs* expression whereas SlPIF4 promotes *CBFs* expression and cold tolerance (Lee and Thomashow 2012; Wang et al. 2020b). Grapevine PIFs have been identified and characterized in *V. vinifera* (Zhang et al. 2018), but the possible roles of VvPIFs in cold response in grapevine remain a subject for further investigation.

### Predominant transcription factors

The APETALA2/ERF (AP2/ERF) family, consisting of AP2, RAV1 (Related to ABI3/VP1), and ERF families, is a key group of TFs in cold response in plants (Ritonga et al. 2021). In a recent study, almost all the identified TFs that respond to cold stress in *V. amurensis* belong to AP2/ERF family (Ren et al. 2021). Overexpression of *VaRAV1* in grape cells enhanced its cold tolerance (Ren et al. 2021). The grapevine VaERF057 and VaERF092 have been demonstrated to positively regulate cold tolerance (Sun et al. 2016, 2019). Additionally, the expression of *VaERF104*, *VaERF1A*, *VaERF115*, and *VaERF4* is also strongly induced by cold, while a *cytokinin response factor 2* (*CRF2*) gene is repressed by cold in *V. amurensis* (Ren et al. 2021). As members of AP2/ERF, CRF2, and CRF3 have been found to participate in root adaptation to cold stress in Arabidopsis (Jeon et al. 2016). The grape AP2/ERF family has already been characterized in *V. vinifera* (Zhuang et al. 2009; Licausi et al. 2010), and expression profiling of grape AP2/ERF genes suggests a specific role for some AP2/ERF members in fruit ripening (Licausi et al. 2010).

The AP2/ERF family could interrelate with other TF families, such as MYB, bHLH (basic helix-loop-helix), WRKY, NAC, and bZIP (basic leucine zipper), to enhance cold tolerance (Ritonga et al. 2021; Bai et al. 2022). The Arabidopsis MYB15 has been reported to be a repressor of *CBFs* expression (Agarwal et al. 2006). In grapevine, the most studied MYB TFs, i.e. MYB14/15 and MYBA1, are involved in biosynthesis of secondary metabolites, such as stilbenes and anthocyanins (Höll et al. 2013; Fang et al. 2014; Wang et al. 2020a; Xie et al. 2020; Cheng et al. 2021). The VaMYB44 negatively regulates cold tolerance in both Arabidopsis and grapevine (Zhang et al. 2022b), while the MYB-like VaAQUILO positively regulates plant cold tolerance (Sun et al. 2018). The ICE1 and PIFs mentioned above belong to the bHLH family. There are 94 bHLH genes in *V. vinifera*, among which 17 genes are induced by cold treatment. Some of the cold-induced genes contain ABA-responsive elements or MYB binding sites in their promoters, suggesting that these genes might be regulated by ABA or MYB (Wang et al. 2018). Moreover, overexpression of *VvbHLH1* or *VabHLH1* could enhance the tolerance of transgenic Arabidopsis to cold stress without affecting plant development (Xu et al. 2014c). In *V. davidii*, however, a total of 115 bHLH genes have been identified, but the functions of *VdbHLHs* in cold response are still unknown (Li et al. 2021a). The WRKY family has been characterized with 59 genes in *V. vinifera* in three independent studies (Guo et al. 2014; Wang et al. 2014a, 2014b). Almost all the *VvWRKY* genes (55 out of 59) could respond to at least one specific abiotic stress (Guo et al. 2014), and cold stress led to rapid up-regulation of *VvWRKY* genes (Wang et al. 2014b). *VvWRKY24* was specifically induced by cold (Wang et al. 2014b), and overexpression of cold-induced *VaWRKY12* and *VpWRKY2* could enhance plant cold tolerance (Li et al. 2010; Zhang et al. 2019). The plant-specific NAC family plays critical roles in plant growth, development, and responses to abiotic stresses (Duan et al. 2017; Diao et al. 2020; Song et al. 2022a). The target genes of NAC TFs in response to abiotic stresses include *CBF3*, *COR15/47*, *RD29* (*responsive to dehydration 29*), *LEA3-1* (*late embryogenesis abundant protein 3-1*), and *GST* (*glutathione S-transferase*) (Puranik et al. 2012; Diao et al. 2020). A total of 74 NAC genes have been identified with distinct expression patterns in different tissues and developmental stages in *V. vinifera* (Wang et al. 2013b). Eight of the 74 *VvNAC* genes are upregulated by cold stress (Wang et al. 2013b). *VvNAC1*-overexpressing Arabidopsis plants show improved tolerance to cold stress and pathogens (Le Hénanff et al. 2013). The involvement of *VvNAC17* in Arabidopsis cold tolerance is also reported recently by upregulating *COR15A*, *COR47*, *RD29A*, and *RD22* genes (Ju et al. 2020). The grapevine bZIP family has been systematically characterized in *V. vinifera* (Liu et al. 2014), but the functions of VvbZIPs in cold response remain largely unknown. Only a bZIP gene named *VvbZIP23* could be induced by cold (Tak and Mhatre 2013). It should be noted that transcriptional regulatory networks in response to cold are sophisticated and flexible, and other TFs, such as early responsive to dehydration (ERD), GRAS, and DNA-binding with one finger (Dof) proteins, are also tightly associated with cold response in grapevine (Yu et al. 2017; Shangguan et al. 2020; Wang et al. 2021d, 2021e).

### Protein kinases and stress-related proteins

Protein kinases (PKs), particularly SnRKs, mitogen-activated protein kinases (MAPKs), calcium-dependent protein kinases (CDPKs/CPKs), and RLKs, are important players in signal transductions of abiotic stresses (Chen et al. 2021). These PKs regulate cold tolerance by post-translational phosphorylation of ICE1–CBF signaling pathway (Shi et al. 2018a; Ding et al. 2019, 2020; Chen *et al*. 2021). The family members of SnRK2s, MAPKs, CDPKs, and RLKs have been identified in grapevine (Di Gaspero and Cipriani 2003; Dubrovina et al. 2013; Zhang et al. 2015, 2022c; Çakır and Kılıçkaya 2015). Among the seven SnRK2 genes, *VvSnRK2.1*, *VvSnRK2.2*, *VvSnRK2.3*, *VvSnRK2.6*, and *VvSnRK2.7* were regulated by chilling stress (Zhang et al. 2022c). For grape *CDPKs*, only a few *CDPK* genes were induced by cold, and *VaCDPK20* was reported to mediate cold and drought tolerance (Zhang et al. 2015; Dubrovina et al. 2015). The roles of these PKs in grapevine cold tolerance remain to be explored.

Protein phosphatases, key enzymes in carbohydrate metabolism, and LEA proteins are all involved in stress responses (De Rosa et al. 2022; Xu et al. 2020). To identify differentially expressed proteins (DEPs) in response to cold stress, comparative proteomic analysis was conducted in grapevine roots, and a total of 25 DEPs covering stress response, metabolism, energy, bio-signaling, and translation were identified to respond to freezing stress (Chen et al. 2022b). Hence, proteomic analysis enables researchers to uncover cold-responsive proteins.

### Noncoding RNAs

Increasing evidence has shown that plant noncoding RNAs (ncRNAs), including small ncRNA (sncRNAs) and long ncRNAs (lncRNAs), play essential roles in cold response. Two recent reviews have discussed the current advances on ncRNAs in cold response in plants (Ma et al. 2022; Huo et al. 2022). Grapevine sncRNAs, including microRNAs (miRNAs) and small interfering RNAs (siRNAs), have been isolated and characterized (Carra et al. 2009; Pantaleo et al. 2010; Wang et al. 2011a, b, 2012; Luo et al. 2018). Most of the identified grapevine miRNAs are involved in grapevine development with tissue-specific expression patterns (Carra et al. 2009; Mica et al. 2010). A total of 44 miRNAs were identified as cold-inducible miRNAs in “Muscat hamburg” by high-throughput sequencing (Sun et al. 2015). These cold-induced miRNAs may regulate cold response by targeting TFs, such as MYB, bHLH, bZIP, and GRAS (Sun et al. 2015). Comparative analysis of cold-related miRNAs in *V. amurensis* and *V. vinifera* uncovered distinct expression patterns of miRNAs in the two species, indicating the existence of different regulatory models of miRNAs in cold response (Wang et al. 2019c). Moreover, species-specific cold-induced miRNAs were also identified (Wang et al. 2019c). These results may partially account for the difference in cold tolerance across different grapevine species. The expression of grapevine lncRNA under cold stress has also been surveyed in *V. vinifera* (Wang et al. 2019b). A great number of known lncRNAs are regulated by cold stress in grapevine, and many differentially expressed lncRNAs are found to target cold-responsive genes, such as *CBF4*, *NACs*, *WRKYs*, and *LEAs* (Wang et al. 2019b). Circular RNAs (circRNAs) are a class of single-stranded RNAs formed by backsplicing. The known functions of circRNAs include modulation of transcription and splicing, interference of mRNAs and protein stability, and even protein translation (Liu and Chen 2022). At present, information about plant circRNAs is very limited. Gao et al. (2019) employed circRNA prediction approaches to identify cold-related circRNAs in grapevine. A total of 475 circRNAs have been identified as cold-responsive circRNAs; among which the *Vv*-*circATS1* derived from *glycerol-3-P acyltransferase* has been demonstrated to enhance cold tolerance in Arabidopsis (Gao et al. 2019). Very recently, two peptides, i.e. vvi-miPEP172b and vvi-miPEP3635b, were applied to grapevine plants to enhance the cold tolerance (Chen et al. 2022a), suggesting a possible role for *vvi-MIR172b* and *vvi-MIR3635b* in regulating cold response in grapevine.

## Approaches to improve cold tolerance in grapevine

Hybridization across different *Vitis* species could reassort genetic variants, especially biotic and abiotic stress-tolerance traits, into beneficial combinations to breed grapevines that are sustainable for production in harsh conditions. The wild *V. amurensis* has been used for breeding with *V. vinifera* to develop interspecific hybrids with preeminent cold tolerance. For instance, the grape varieties “Beihong” and “Beimei”, two hybrids of *V. amurensis* and “Muscat humberg” (*V. vinifera*), withstand LTs below −20°C (Chai et al. 2015). The genes involved in cold tolerance in *V. amurensis* could be introduced into offspring through hybridization. However, information on these genes is still lacking. Genome assembly of *V. amurensis* uncovered gene expansion that contributes to cold tolerance, and genome-wide association study revealed a phosphoglycerate kinase gene that may be associated with freezing tolerance in grapevine buds (Wang et al. 2021c). Cutting-edge sequencing technologies bring us increasing number of grapevine genomes, resequencing and transcriptome data (Xin et al. 2013; Xu et al. 2014b; Londo et al. 2018; Girollet et al. 2019; Liang et al. 2019; Patel et al. 2020; Morales-Cruz et al. 2021), which would facilitate the development of molecular markers through population-based genomics. It would be meaningful to introgress genes of interest (e.g. cold tolerance) into specific cultivars through marker-assisted backcrossing. However, though plenty of markers could be generated through next generation sequencing-based genetic mapping, there are still many difficulties that hinder the application of molecular markers (Ribaut et al. 2010; Yang et al. 2015). With respect to breeding of cold-hardy grapevines, e.g. it is unclear what criteria should be adopted for marker development and whether the developed marker could be applied in breeding practice. Recently, transcriptomic analysis combined with ATAC-seq (assay for transposase-accessible chromatin with sequencing) has been successfully used to identify cold-responsive TFs in grapevine (Ren et al. 2021). It provides a promising strategy for mining candidate genes for improvement of grapevine cold tolerance through genetic engineering. Moreover, genome editing technologies could be employed to decipher the functions of genes of interest and improve cold tolerance of grapevines in a designed manner.

Grafting is an important agronomic technology to improve the quality traits and/or tolerance of scions to environmental stresses. Enhanced cold tolerance of grafted seedlings has been accomplished recently in watermelon (*Citrullus lanatus*) and cucumber (*Cucumis sativus*), respectively (Lu et al. 2022; Sun et al. 2022). Actually, grafting with grapevine rootstocks could promote expression of numerous TFs and accumulation of stilbene, flavonol, and ABA in scions (Chitarra et al. 2017; Zhang et al. 2022a). Therefore, grafting may serve as an approach to improve cold tolerance in grapevine.

As mentioned above, application of synthetic peptides encoded by primary transcript of miRNAs (miPEPs) could increase cold tolerance of grapevine plants (Chen et al. 2022a), which provides a strategy for cold tolerance improvement in grapevine. Hence, it would be fruitful to probe miPEPs involved in cold tolerance in grapevine. Epiphytic or endophytic plant growth-promoting rhizobacteria (PGPR) could enhance plant growth and resistance to abiotic stresses by affecting nutrient and water management and phytohormone production (Dobbelaere et al. 2003; Compant et al. 2005; Li et al. 2022). Inoculation of grapevine plants with the *Burkholderia phytofirmans* strain PsJN remarkably improves plant cold tolerance, with increased levels of proline, phenolics, and starch (Barka et al. 2006). In addition to the accumulation of carbohydrates, PsJN-bacterized plants accumulate trehalose and trehalose-6-phosphate in stems and leaves, and net photosynthesis is also less affected (Fernandez et al. 2012a, b; Theocharis et al. 2012). Interestingly, stress-related genes in PsJN-bacterized plants are induced more rapidly and earlier after exposure to cold (Theocharis et al. 2012). These results suggest that the use of PGPR may be a useful technology to protect grapevine from cold-induced damage.

### Concluding remarks

LTs pose a major threat to grapevine growth and production. The cultivated *V. vinifera* is generally sensitive to LTs, especially freezing stress, which is commonly encountered by vines in winter in North China. Wild species like *V. amurensis* exhibit strong cold tolerance, but the underlying mechanisms remain largely unknown. To identify quantitative trait loci or variations involved in cold tolerance would promote our understanding of cold resistance and also the breeding of cold-hardy grapevines. Moreover, though great advances have been achieved in model plants, our knowledge of the molecular mechanisms of cold response in grapevine is still fragmented, and whether the knowledge from other species could be applied in grapevine remains to be determined. Furthermore, how to use our knowledge of cold-regulatory mechanisms to develop elite grapevines with enhanced cold tolerance for growth in the field remains a big challenge (see “Outstanding Questions”). Crossbreeding, grafting with cold-tolerant rootstocks, inoculation of beneficial bacteria, and application of synthetic peptides could be employed to improve cold tolerance in viticulture. Given that adverse temperatures usually attenuate plant growth and production, breeding grapevines with improved abiotic stress tolerance and high fruit quality for adaption to global climate changes would be an important issue in the future.

**Outstanding questions Box**

- What are the key genes underlying the strong freezing tolerance in the wild species *V. amurensis*?
- As conventional breeding remains the leading approach for creation of desirable inherited traits, how can we retain the desired cold-related genes in offspring during recurrent backcrossing?
- How can we utilize basic knowledge of cold-responsive mechanisms obtained in the laboratory to develop cold-tolerant grapevines for growth in the field?
- How can we orchestrate abiotic stress tolerance (e.g. cold and drought) and grapevine growth (e.g. production and fruit quality) in harsh conditions?
